# Emergence of mobile tigecycline resistance mechanism in *Escherichia coli* strains from migratory birds in China

**DOI:** 10.1080/22221751.2019.1653795

**Published:** 2019-08-20

**Authors:** Chong Chen, Chao-Yue Cui, Yan Zhang, Qian He, Xiao-Ting Wu, Gong Li, Xiao-Ping Liao, Barry N. Kreiswirth, Ya-Hong Liu, Liang Chen, Jian Sun

**Affiliations:** a National Risk Assessment Laboratory for Antimicrobial Resistance of Animal Original Bacteria, College of Veterinary Medicine, South China Agricultural University, Guangzhou, People’s Republic of China; b Hackensack-Meridian Health Center for Discovery and Innovation, Nutley, NJ, USA

**Keywords:** Tigecycline resistance, migratory birds, *tet*(X4), eravacycline, omadacycline

## Abstract

Plasmid-mediated antimicrobial resistance has emerged as one of the principal global issues, posing significant threats to public health. Herein, we reported a mobile tigecycline resistance mechanism Tet(X4) on both plasmid and chromosome in *Escherichia coli* strains from migratory birds in China. Besides tigecycline, these *tet*(X4)-positive strains also exhibited elevated MICs to the FDA newly approved tetracycline antibiotics, eravacycline (4 µg/ml) and omadacycline (8 µg/ml). Worrisomely, the *tet*(X4)-carrying plasmids and chromosome also shared high homology with the plasmids from human. Taken together, Tet(X4) represents another emerging antimicrobial threat and collective efforts from different sectors are needed to control its further spread.

Recent studies have showed that wild birds, especially migratory birds, may play an important role in the emergence and transmission of antimicrobial resistance and infectious diseases [1]. Meanwhile, the tigecycline resistance has also sporadically occurred (although not in migratory birds), primarily due to overexpression of efflux pumps and the newly identified tigecycline-inactivating mechanisms Tet(X3) and Tet(X4) [2–4]. Here we reported the *tet*(X4) gene in *Escherichia coli* strains from migratory birds in China, on both plasmid and chromosome, which raised a serious public health concern.

In 2018, three tigecycline-resistant *E. coli* strains, namely 2FT39, 2FT38-2, and 2ZN37-2, were isolated from stool samples of the little egrets (*Egretta garzetta*) from Guangdong, China, during a wild bird antimicrobial resistance surveillance study (Table S1). Susceptibility testing results showed that they were all resistant to tigecycline, tetracycline, florfenicol, and sulfamethoxazole-trimethoprim (Table S2). In addition, *E. coli* 2FT39 was also resistant to ciprofloxacin and *E. coli* 2FT38-2 was resistant to gentamicin. Interestingly, the three *E. coli* strains also exhibited significantly higher minimal inhibitory concentrations (MICs) to the FDA newly approved tetracycline antibiotics, eravacycline (4 µg/ml) and omadacycline (8 µg/ml), in comparison to the results from previous surveillance studies [5,6].

Further conjugation experiments by filter mating showed that the tigecycline resistance could be successfully transferred from *E. coli* 2FT38-2 into the recipient *E. coli* C600, *Salmonella Typhimurium* ATCC 14028 and clinical KPC-2-producing *Klebsiella pneumoniae* 1332, with the transfer efficiencies of (4.3 ± 0.7)×10^−2^, (1.2 ± 0.4)×10^−6^ and (1.6 ± 0.5)×10^−4^, respectively. Meanwhile, the resistance to tetracycline, florfenicol, sulfamethoxazole-trimethoprim and gentamicin were co-transferred (Table S2). The transconjugants also exhibited at least 8-fold increases of MICs against eravacycline and omadacycline, in comparison to the recipients. By contrast, the tigecycline resistance in *E. coli* 2FT39 failed to transfer via conjugation, but was successfully transferred into *E. coli* JM109 by electroporation, along with tetracycline and florfenicol resistance (Table S2). Similarly, the 2FT39 transformant had 512- and 32-fold increases of MICs against eravacycline (4 µg/ml) and omadacycline (8 µg/ml), respectively. However, the tigecycline resistance in *E. coli* 2ZN37-2 failed to transfer either by conjugation or electroporation.

Genomic DNA of them was then completely sequenced by combination of Nanopore GridION and Illumina HiSeq platforms (Nextomics, Wuhan, China), followed by assembling with Unicycler and annotating with the RAST server [7,8]. Subsequently, the bioinformatics analyses of them were conducted via the CGE server (https://cge.cbs.dtu.dk/services/), with ResFinder for detection of antimicrobial resistance genes, Multi-Locus Sequence Typing (MLST) for sequence types (STs), and PlasmidFinder for plasmid replicon types [9–11]. *E. coli* 2FT39 (Accession number: SSWK00000000), 2FT38-2 (SSWJ00000000), and 2ZN37-2 (SSWI00000000) all harbored a gene [namely *tet*(X4)] homologous to *tet*(X) with 94.5% amino acid sequence identity, which has been confirmed to share the similar degradation mechanism for tetracyclines (including tigecycline) by hydroxylation at C11a [4]. In this study, further gene cloning of *tet*(X4) from 2ZN37-2 into a pBAD24 vector also showed 64-, 512-, and 32-fold increases of MICs for tigecycline (16 µg/ml), eravacycline (4 µg/ml) and omadacycline (8 µg/ml), respectively.

The MLST analysis showed that the *E. coli* 2FT39, 2FT38-2, and 2ZN37-2 strains were non-clonal and belonged to three distinct STs: ST1196, ST6833, and ST641 (Table S1). Especially, the *tet*(X4) gene was identified on two different plasmids (namely p2FT39-3 and p2FT38-2-1) in strain 2FT39 and 2FT38-2, and on the chromosome of strain 2ZN37-2 (c2ZN37-2), respectively. These were further confirmed by S1-PFGE, I-*Ceu*I PFGE, and Southern blot hybridization (Figure S1 and S2).

Among them, p2FT39-3 was 68.714 kb in size and harbored 75 open reading frames (ORFs), including *tet*(X4), *erm*(42) and *floR* (Figure 1(A)). Plasmid structural analysis showed that it belonged to the F-:A18:B- plasmid group but lacked the entire conjugative transfer region, which could explain its failure in the conjugation experiment. Blast analysis showed that p2FT39-3 shared similar plasmid backbone (70% query coverage and 99% average sequence identity) against some other plasmids deposited in GenBank, such as p14EC001c (CP024130) from a clinical *E. coli* strain and p3_W5-6 (CP032995) from an *E. coli* strain isolated from a contaminated waterway of wild birds.

**Figure 1.**
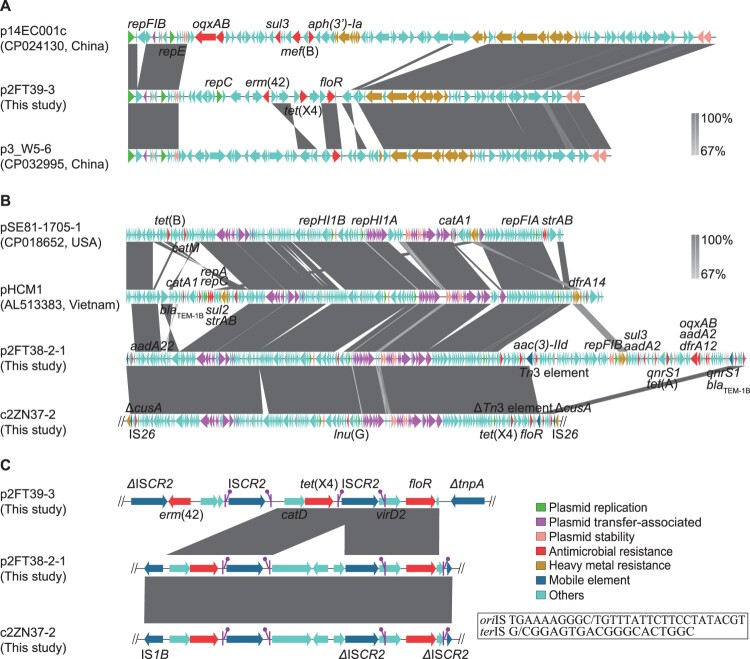
Comparative analysis of *tet*(X4)-harboring plasmids and chromosome. (A) Structures of p14EC001c (CP024130), p3_W5-6 (CP032995) and the *tet*(X4)-harboring plasmid p2FT39-3. (B) Structures of pSE81-1705-1 (CP018652), pHCM1 (AL513383), the *tet*(X4)-harboring plasmid p2FT38-2-1 and *tet*(X4)-harboring chromosome region in *E. coli* 2ZN37-2. (C) Genetic environments of *tet*(X4). Results of nucleotide sequence alignment are generated with Easyfig [15]. The arrows represent the positions and transcriptional directions of the ORFs. Regions of homology are marked by shading. The putative *ori*IS (left-facing sticks in purple) and *ter*IS (right-facing sticks in purple) of the IS*CR2* element are also illustrated (5′ to 3′). Δ symbol indicates that the gene is truncated.

p2FT38-2-1 was 283.255 kb in length and contained 349 ORFs, including the resistance genes *aadA2*, *aac(3)-Iid*, *aadA22*, *bla*_TEM−1B_, *qnrS1*, *oqxAB*, *lnu*(G), *floR*, *sul3*, *tet*(A), *dfrA12* and *tet*(X4) (Figure 1(B)). Plasmid structural analysis showed that it belonged to IncHI1 incompatibility group, harboring an intact conjugation transfer region, which was in concordance with its conjugability described above. Interestingly, p2FT38-2-1 also harbored partial skeleton of the F-:A8:B- plasmid, including plasmid replication, transfer and stability regions, and was similar to the plasmids pSE81-1705-1 (CP018652) and pHCM1 (AL513383) in *Salmonella* spp. from human samples.

The *tet*(X4) gene in *E. coli* 2ZN37-2 was identified in a 194.2 kb genomic island on its chromosome (4931.759 kb), along with some other antimicrobial resistance genes, including *aadA22*, *bla*_TEM−1B_, *qnrS1*, *lnu*(G), and *floR* (Figure 1(B)). This genomic island was flanked by two copies of IS*26* and inserted into a heavy metal resistance gene *cusA*, with high homology (99%) to the sequence of p2FT38-2-1, likely as a result of IS*26*-mediated genetic element mobilization. Similar insertion sequence-mediated chromosome integrations have also been described in other resistance genes, including *aphA1*, *tet*(D) and *bla*_SHV_ [12].

We then examined the *tet*(X4)-neighboring genetic elements among the three strains, and found that *tet*(X4) was located upstream of IS*CR2* and downstream of a hydrolase gene *catD* (Figure 1(C)). Unlike other insertion sequences, a single copy of IS*CR2* could utilize a rolling-circle transposition process to transpose adjacent DNA sequences [13]. It has been found to be associated with the acquisition of diverse antimicrobial resistance genes, including *bla*_VEB−1_ [14]. Interestingly, the *catD*-*tet*(X4) element in p2FT39-3 was found be flanked by two copies of IS*CR2*, which strongly suggested that IS*CR2* was associated with the mobilization of *tet*(X4). It was likely that an intact IS*CR2* copy originally mobilized *catD*-*tet*(X4) by a rolling-circle transposition process and that a secondary process of homologous recombination between two IS*CR2* copies led to the deletion of one copy of IS*CR2*. In combination with the conjugability observed in p2FT38-2-1, our results also suggested that the *tet*(X4) gene might be able to transfer into other plasmid vectors and spread into other bacterial strains.

In summary, we reported the emergence of *tet*(X4)-encoding tigecycline resistance mechanism in *E. coli* strains from migratory birds, further highlighting that migratory birds may serve as a reservoir for the dissemination of emerging antimicrobial resistance. Worrisomely, the emergence of *tet*(X4) challenges the effectiveness of the entire family of tetracycline antibiotics, including the FDA newly approved eravacycline and omadacycline. Further spread of *tet*(X4) into clinical multidrug-resistant pathogens may create extensively drug-resistant or pandrug-resistant strains, and result in untreatable infections. A continuous surveillance of *tet*(X4) in humans, animals, and their environments should be considered for understanding and tackling the dissemination of tigecycline resistance.

## Geolocation information

These *tet*(X4)-positive *E. coli* strains were isolated from migratory birds in Shenzhen (*E. coli* 2FT39; *E. coli* 2FT38-2) and Huizhou (*E. coli* 2ZN37-2), China, respectively.

## Acknowledgements

JS, LC, Y-HL, and X-PL designed the study. CC, C-YC, YZ, QH, X-TW, and GL collected the samples and conducted the experiments. JS, LC, and CC analyzed and interpreted the data. JS, LC, BNK, and CC drafted the manuscript. All authors reviewed, revised, and approved the final report.

## Supplementary Material

Supplemental Material
