# Supplementary material for: Emergence of mobile tigecycline resistance mechanism in Escherichia coli strains from migratory birds in China
Source: Emerg Microbes Infect. 2019 Aug 20;8(1):1219–22. doi: 10.1080/22221751.2019.1653795 (PMC6713155; doi:10.1080/22221751.2019.1653795)
Supplement: Supplemental Material [file TEMI_A_1653795_SM4605.zip › Supplementary_Information.docx]

**Supplementary Information for**

**Title: Emergence of mobile tigecycline resistance mechanism in *Escherichia coli* strains from migratory birds in China**

Chong Chen^1^, Chao-Yue Cui^1^, Yan Zhang^1^, Qian He^1^, Xiao-Ting Wu^1^, Gong Li^1^, Xiao-Ping Liao^1^, Barry N. Kreiswirth^2^, Ya-Hong Liu^1^, Liang Chen^2*^ and Jian Sun^1*^

^1^National Risk Assessment Laboratory for Antimicrobial Resistance of Animal Original Bacteria, College of Veterinary Medicine, South China Agricultural University, Guangzhou, China.

Chong Chen, E-mail: 1634664169@qq.com

Chao-Yue Cui, E-mail: c18320721812@163.com

Yan Zhang, E-mail: 978946021@qq.com

Qian He, E-mail: 670146947@qq.com

Xiao-Ting Wu, 946811334@qq.com

Gong Li, E-mail: 1034126372@qq.com

Xiao-Ping Liao, E-mail: xpliao@scau.edu.cn

Ya-Hong Liu, E-mail: lyh@scau.edu.cn

Jian Sun, E-mail: jiansun@scau.edu.cn

^2^Hackensack-Meridian Health Center for Discovery and Innovation, Nutley, NJ, USA.

Barry N. Kreiswirth, E-mail: barry.kreiswirth@hmh-cdi.org

Liang Chen, E-mail: Liang.Chen@hmh-cdi.org

^*^Corresponding authors

Jian Sun PhD: College of Veterinary Medicine, South China Agricultural University, Guangzhou 510642, China. E-mail: [jiansun@scau.edu.cn](mailto:xpliao@scau.edu.cn). Tel: +86-020-85285507; Fax: +86-020-85285507.

Liang Chen PhD: Hackensack-Meridian Health Center for Discovery and Innovation, Nutley, NJ, USA. E-mail: Liang.Chen@hmh-cdi.org. Tel: +201-880-3522; Fax: +201-880-3522.

**Table S1.** Characteristics of *tet*(X4)-positive *E. coli* isolates from migratory birds in China.

| Strain | Date | Isolation site | MLST | Location of *tet*(X4) | |
| --- | --- | --- | --- | --- | --- |
|  |  |  |  | Size (kb) | PBRT |
| 2FT39 | Jan 2018 | Shenzhen, Guangdong | ST1196 | 68.714 | F-:A18:B- |
| 2FT38-2 | Jan 2018 | Shenzhen, Guangdong | ST6833 | 283.255 | IncHI1-F-:A8:B- |
| 2ZN37-2 | Mar 2018 | Huizhou, Guangdong | ST641 | 4931.759 | ND^a^ |

^a^ND, not detected. The *tet*(X4) gene in *E. coli* 2ZN37-2 is chromosome-derived and has no replicon type.

**Table S2.** MICs of the parental strains, transconjugants and transformants.

| Strain | Source | MICs (ug/ml)^a^ | | | | | | | |
| --- | --- | --- | --- | --- | --- | --- | --- | --- | --- |
|  |  | TGC | TC | FFC | SXT | CIP | GEN | ERA | OMA |
| *E. coli* 2FT39 | Egret | 8 | 128 | 256 | >320 | 256 | 8 | 4 | 8 |
| *E. coli* JM109 | Laboratory strain | 0.13 | 1 | 2 | 2.5 | 0.03 | 0.5 | 0.008 | 0.25 |
| *E. coli* JM109+p2FT39-3 | Transformant | 8 | 64 | 128 | 2.5 | 0.03 | 0.5 | 4 | 8 |
| *E. coli* 2FT38-2 | Egret | 8 | 128 | 256 | >320 | 2 | 64 | 4 | 8 |
| *E. coli* C600 | Laboratory strain | 0.03 | 0.5 | 2 | 2.5 | 0.03 | 1 | 0.008 | 0.13 |
| *E. coli* C600+p2FT38-2-1 | Transconjugant | 4 | 64 | 128 | >320 | 0.06 | 64 | 2 | 4 |
| *S. Typhimurium* ATCC 14028 | Laboratory strain | 0.13 | 1 | 4 | 5 | 0.03 | 0.25 | 0.13 | 0.5 |
| *S. Typhimurium* ATCC 14028+p2FT38-2-1 | Transconjugant | 8 | 128 | 256 | >320 | 0.03 | 64 | 4 | 16 |
| *K. pneumoniae* 1332 | Patient | 1 | 4 | 256 | >320 | 64 | >256 | 0.5 | 2 |
| *K. pneumoniae* 1332+ p2FT38-2-1 | Transconjugant | 16 | 256 | 256 | >320 | 64 | >256 | 8 | 16 |
| *E. coli* 2ZN37-2 | Egret | 8 | 128 | 256 | >320 | 2 | 1 | 4 | 8 |

^a^TGC, tigecycline; TC, tetracycline; FFC, florfenicol; SXT, sulfamethoxazole-trimethoprim; CIP, ciprofloxacin; GEN, gentamicin; ERA, eravacycline; OMA, omadacycline.


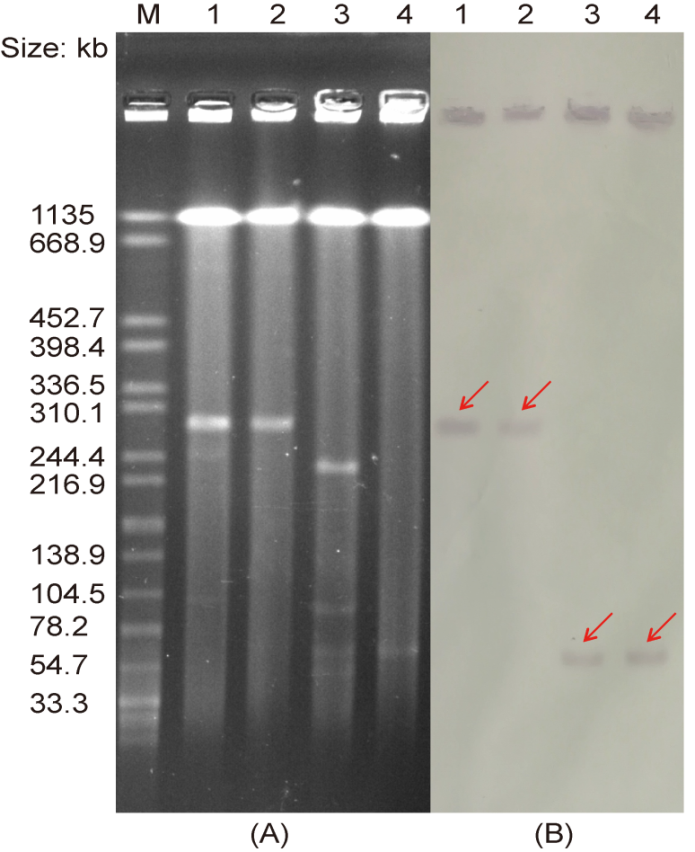


**Figure S1.** The localization of *tet*(X4) by S1-PFGE and Southern blot hybridization. (A) PFGE profiles of S1-digested total DNA. (B) Hybridization results with the probe for *tet*(X4). M, *S. enterica* serotype Braenderup strain H9812 DNA digested with *Xba*I, used as a molecular marker (kb). Lanes: 1, *E. coli* 2FT38-2; 2, *E. coli* C600+p2FT38-2-1; 3, *E. coli* 2FT39; 4, *E. coli* JM109+p2FT39-3.


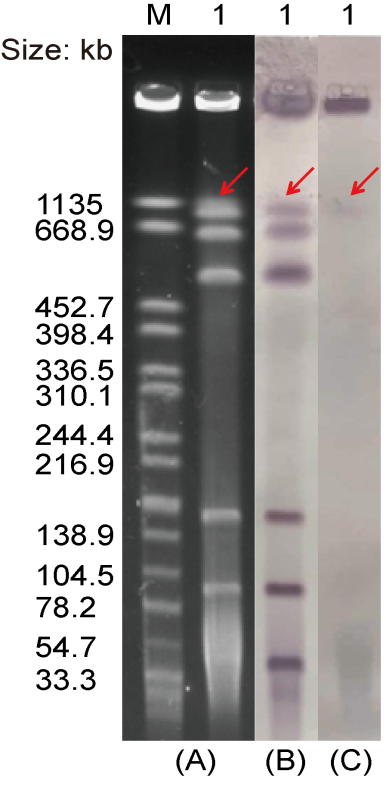


**Figure S2.** The localization of *tet*(X4) by I-*Ceu*I PFGE and Southern blot hybridization. (A) PFGE profiles of I-*Ceu*I digested total DNA. (B) Hybridization result with the probe for 23S rDNA. (C) Hybridization result with the probe for *tet*(X4). The result showed that the *tet*(X4) is located on the same chromosome band of 23S rDNA. Lanes: M, H9812 marker (kb); 1, *E. coli* 2ZN37-2.
